# Using Animal Performance Data to Evidence the Under-Reporting of Case Herds during an Epizootic: Application to an Outbreak of Bluetongue in Cattle

**DOI:** 10.1371/journal.pone.0100137

**Published:** 2014-06-17

**Authors:** Simon Nusinovici, Pascal Monestiez, Henri Seegers, François Beaudeau, Christine Fourichon

**Affiliations:** 1 INRA, UMR1300 Biology, Epidemiology and Risk Analysis in animal health, Nantes, France; 2 LUNAM Université, Oniris, Ecole nationale vétérinaire, agroalimentaire et de l’alimentation Nantes-Atlantique, Nantes, France; 3 INRA, UR 546, Biostatistics and Spatial Processes, Avignon, France; The Pirbright Institute, United Kingdom

## Abstract

Following the emergence of the Bluetongue virus serotype 8 (BTV-8) in France in 2006, a surveillance system (both passive and active) was implemented to detect and follow precociously the progression of the epizootic wave. This system did not allow a precise estimation of the extent of the epizootic. Infection by BTV-8 is associated with a decrease of fertility. The objective of this study was to evaluate whether a decrease in fertility can be used to evidence the under-reporting of cases during an epizootic and to quantify to what extent non-reported cases contribute to the total burden of the epizootic. The cow fertility in herds in the outbreak area (reported or not) was monitored around the date of clinical signs. A geostatistical interpolation method was used to estimate a date of clinical signs for non-reported herds. This interpolation was based on the spatiotemporal dynamic of confirmed case herds reported in 2007. Decreases in fertility were evidenced for both types of herds around the date of clinical signs. In non-reported herds, the decrease fertility was large (60% of the effect in reported herds), suggesting that some of these herds have been infected by the virus during 2007. Production losses in non-reported infected herds could thus contribute to an important part of the total burden of the epizootic. Overall, results indicate that performance data can be used to evidence the under-reporting during an epizootic. This approach could be generalized to pathogens that affect cattle’s performance, including zoonotic agents such as *Coxiella burnetii* or Rift Valley fever virus.

## Introduction

Following the emergence of the Bluetongue virus serotype 8 (BTV-8) in France in 2006, a surveillance system was implemented to detect and follow precociously the progression of the epizootic wave. This system was composed of both passive (detection of clinical signs of the disease by farmers) and active surveillance (blood sampling and diagnostic tests in targeted populations). This system did not allow a precise estimation of the extent of the epizootic. A cross-sectional serologic study conducted in 2007 showed that only a low proportion of seropositive herds reported clinical cases, indicating a high under-reporting rate of clinical cases [1]. Several reasons could explain the fact that some herds infected by BTV-8 were not reported during the epizootic. Firstly, because most infected cattle herds showed no evidence of clinical signs, their infection was not systematically noticed by farmers and veterinary practitioners [1], [2], [3]. Secondly, the fact that BTV-8 recently emerged in this part of Europe complicated the identification of the disease because of the low level of awareness (only 6 case herds were reported in 2006 in mainland France). Therefore, even in case of clinical expression of the disease, farmers could not have detected them. Moreover, they could have detected clinical signs but not notified them. As suggested, there is some reluctance to report suspect clinical situations in general by farmers and veterinary practitioners to the veterinary authorities in fear of anticipated social and economic consequences [3]. Finally, most clinical signs of the disease are not specific, and therefore could have been detected but not attributed by farmers to BTV-8 infection. Concerning the active surveillance system, the objective was to detect the virus in areas previously free of virus, using very small samples. The surveillance was stopped in a given area once the virus was detected. This system was thus not designed to quantify the incidence of the infection. These facts highlight the limits of surveillance systems performance in the event of an emerging disease.

Infection by BTV-8 is associated with a decrease of fertility [4], [5]. For cows in herds reported after clinical signs suspicion during the BTV-8 epizootic in 2007 in France, fertility was adversely affected around the date of detection of clinical signs in the herd [5]. A decreased fertility can be expected for cows in herds not reported during the epizootic, if a proportion of these herds are in fact infected. It can be assumed that the magnitude of the decrease in undetected herds depends on the proportion of herds that were infected. The magnitude is likely to be lower compared to the variations quantified in reported herds.

In most dairy cattle, artificial inseminations (AI) are used for reproduction. This information on individual cow reproduction is centralized in a national data base. The monitoring of these events can be used to evaluate reproduction performance at the cow level. For example, the occurrence of repeated AI can be used as proxy of cow fertility disorders [6], [4], [7], [8].

Decrease in fertility could be used as non-specific indicator to evaluate the existence of under-reporting of BTV-8 cases in exposed areas. Moreover, quantifying such losses in non-reported herds would enable to obtain a more comprehensive evaluation of the burden of an epizootic in a newly infected area than when accounting only for case herds. The objective of this study was to evaluate whether a decrease in fertility can be used to evidence the under-reporting of cases during an epizootic and to quantify to what extent non-reported cases contribute to the total burden of the epizootic.

## Materials and Methods

### General Study Design and Available Data

Decrease in fertility was quantified for cows in reported case herds and cows in non-reported herds located in the 2007 outbreak area (herds with uncertain infectious status). These quantifications were performed using cows in herds unexposed to BTV-8 in 2007 as reference population.

Information about exposure of herds to BTV-8 during 2007 was obtained from the official veterinary surveillance system. Herds were reported in 2 distinct situations: (i) in the event of clinical signs detected by the farmer or the veterinary practitioner, subsequently confirmed by a diagnostic test and (ii) in the event of a positive serological test performed either before animal transfer or sale, or in sentinel herds. Among herds reported during 2007, only herds with a confirmed detection reported after clinical suspicion (situation (i)) were included as the date of a positive serological test did not necessarily identify the possible date of BTV-8 exposure of the herd. Information about BTV-8 exposure was available at the herd level only. Thus, a herd was considered exposed if at least 1 animal with clinical signs had tested positive for BTV-8. The proportion of infected animals in reported herds was unknown. Herds reported after clinical suspicion and subsequently selected for this study will be referred to as ‘case herds’. Case herds located in 23 departments were selected (n = 8,494), corresponding to 96% of all cattle herds reported due to clinical suspicion during the 2007 epizootic. Cattle herds that were not reported during 2007 and located in these departments were considered to have an uncertain infectious status, and were thus referred as ‘herds with uncertain BTV-8 status’ (n = 46,569).

The performance data were obtained in dairy herds enrolled in the official Milk Recording Scheme and where artificial insemination was used. For each cow, obtained data were dates of AIs, rank of service (number of AI within lactation), date of culling (if it had occurred during the study period) and data used to adjust for factors known to influence fertility: cow and bull breeds, lactation number, calving date, milk production data at each milk record (record date, milk yield, protein content, fat content). After selection of dairy herds that use AI for reproduction, populations of case herds and herds with uncertain BTV-8 status were composed of 4,392 and 13,804 herds respectively.

A date of exposure to BTV-8 was either estimated from recorded data for cows in case herds, or interpolated for cows in herds with uncertain BTV-8 status. This interpolation was based on the spatio-temporal dynamics of detection of confirmed case herds that reported clinical signs in 2007. Decrease in fertility in both case herds and herds with uncertain BTV-8 status were quantified around the date of exposure (observed or predicted). The geographical coordinates of herds were available at the municipality level.

### Estimated Date of Exposure for Reported Case Herds

For each case herd, available data included the date at which clinical signs of disease were first suspected and the date at which disease was confirmed via diagnostic tests. The estimated date of exposure for reported herds was defined as the recorded date of suspicion which corresponded to the first detection of clinical signs in the herd. The same date of exposure was assigned to all cows in a herd. For 6.1% of the case herds, the date of clinical suspicion was missing but the date of confirmation by a diagnostic test was known. In order to assign a date of suspicion, an imputation method based on the distribution of the time interval between dates of suspicion and confirmation was applied (values selected at random around a median interval of 4 days). Moreover, 181 case herds that had a non-plausible time interval between dates of suspicion and confirmation (interval >30 days or date of suspicion posterior to the date of confirmation) were excluded.

### Interpolation of a Date of Detection of Clinical Signs for Herds Not Reported and Located in Exposed Areas

A date of exposure to BTV-8 for each cattle herd with uncertain BTV-8 status during 2007 was interpolated.

Kriging, a geostatistical interpolation method, was used to estimate a date of detection of clinical signs for herds with uncertain BTV-8 status. Dates were expressed as the number of days since the first case herd reported in 2007. Kriging uses data sample (cattle case herds) to predict values at unsampled locations (herds with uncertain BTV-8 status). All the cattle herds (dairy and beef) were included because they could have all played a role in the epizootic wave diffusion. Both beef and dairy herds are exposed to *Culicoïdes*. Densities of beef and dairy herds widely vary among French areas. This method is based on assumptions regarding the form of the trend of the sample data, its variance and spatial correlation. The first step consisted in modelling the spatial correlation of the data. Two models were compared. A cross-validation process with observed data was used to determine each model’s goodness of fit and to compare their predictions. Spatial variation in detection dates were modelled using a gaussian semivariogram – for smooth long-range propagation waves - and an exponential semivariogram - for short-range random propagation between neighbouring herds - models. To account for the non stationarity of the BTV-8 spreading process, the gradient of the viral diffusion was also included in the model by the use of Universal Kriging in place of Ordinary Kriging. For the final interpolation of detection dates, only the gaussian-model spatial component was kept for filtering the random local component.

### Selection of Unexposed Herds and Cows

A reference population was used to quantify both decrease in fertility of cows in case herds and cows in herds with uncertain BTV-8 status. It was composed of cows located in 2 French regions unexposed to BTV-8 during 2007: Brittany and a south-western area. In France, herd management varies between areas. Therefore, unexposed cows from 2 different parts of France were selected in order to better represent the unexposed population. This comparison limited the impact of any possible confounding factors due to variations of herd management over time.

The month of AI is known to influence fertility [7], [8]. Consequently, unexposed cows were selected according to the date of AI such that cows from exposed herds and unexposed cows underwent AI during the same period in the year.

### Fertility Parameter and Data Selection

Fertility was assessed by the occurrence of a repeat AI after the first AI. In case of a return-to-oestrus after the first AI in a lactation, most farmers are likely to decide to reinseminate the cow. In such a context, the occurrence of a repeat AI (return-to-service) after a first AI can be used as a proxy for infertility. The criteria used to quantify effect of BTV-8 exposure on fertility was the 90-d-return-to-service, defined as a return-to-service occurring between 18 and 90 d after AI (binary variable). It was assumed that BTV-8 exposure could have 3 possible effects on fertility: conception failure, embryonic death and fetal death in early gestation, all resulting in the same outcome. Cows with non-plausible or extreme data (checked for consistency of calving to first AI interval, AI to calving interval, interval between AI, interval between 2 test-days, peak milk yield and minimum Protein:Fat ratio) and herds with unusual management (unusual demographic structure, delayed first service or very small herds, low rate of IA indicative of the use of a bull) were excluded. After these exclusions, the study population was composed of 122,079 cows with a first AI performed in 2007 (43,786 cows in case herds and 78,293 in herds with uncertain BTV-8 status) in 7,883 herds located in the epizootic area (2,646 case herds and 5,237 herds with uncertain BTV-8 status). The reference population was composed of 211,578 cows in 9,485 herds located in regions unexposed to BTV-8.

### Selection and Classification of AIs to Evaluate the Under-reporting

To evaluate the under-reporting of BTV-8 exposure, 90-d-return-to-service of cows in case herds and cows in herds with uncertain BTV-8 status were compared to those of cows in the reference population. All first AIs performed from 10 weeks before to 14 weeks after the date of BTV-8 exposure (observed or predicted) in herds were selected. The AIs were classified into categories according to the time interval between the date of the AI of cows and the date of exposure (observed or predicted). Intervals of 15 d were considered (see results for categories).

### Statistical Models

The relationship between exposure and occurrence of a possible return-to-service was assessed with multivariate Cox models. To account for factors likely to influence the probability of return-to-service, the association between BTV-8 exposure and occurrence of return-to-service was adjusted for several independent variables already described as risk factors for fertility traits in the literature [6], [7], [8], [9], [10] as described by Eq. (1):
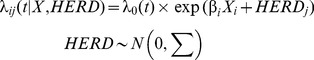
(1)where 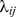 is the hazard function at time *t* for the probability of 90-d-return-to-service following the first AI for the i^th^ cow in the j^th^ herd, X is the vector of the 6 following fixed effect variables: the exposure status (36 classes corresponding to 12 periods of 2 weeks for 3 populations); the lactation number (4 classes)_;_ the maximum milk production in kg at the 3 first milk records in the lactation used as a proxy for peak milk yield (5 classes)_;_ the minimum of Protein:Fat ratio out of the first 3 milk records (5 classes)_;_ the calving-to-AI interval (7 classes) and the month of AI (8 or 10 classes), 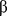 is a vector which contains the coefficients for the 6 fixed effect variables and HERD is the random effect term corresponding to the herd number which follow a normal distribution (0,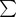). The random effect term made it possible to adjust for clustering within the data using frailty model. The effects in percentage points of return rate were calculated from estimated hazard ratio (HR). All statistical analyzes were performed by using R software [11], Cox models were performed using the survival package (Therneau T, 2008. A package for survival analysis in S) and kriging map using gstat package (Pebesma, EJ, 2004. Multivariable geostatistics in S).

### Selection of a Cow Population to Check the Specificity of the Fertility Decreases

To check whether decreases in fertility in both case herds and herds with uncertain BTV-8 status can be attributable to BTV-8 exposure, possible decrease was also quantified for cows in herds that were not exposed to BTV-8. Indeed, decrease could be related to the climate or diet. This population was considered to demonstrate the specificity of the fertility decreases regarding the BTV-8 exposure, reported or not. This population was composed of cows inseminated in 2005 in herds that were located in the 2007 outbreak area (reported or not). The date around which fertility decreases were quantified corresponded to the same day within the year (called ‘transposed date’) than the date of exposure (observed or predicted). This population (after the same exclusions than the exposed and unexposed populations) was composed of 211,578 cows with a first AI in 9,485 herds.

## Results

### Location of Herds and Unadjusted Return-to-service Rates

**Figure 1:**
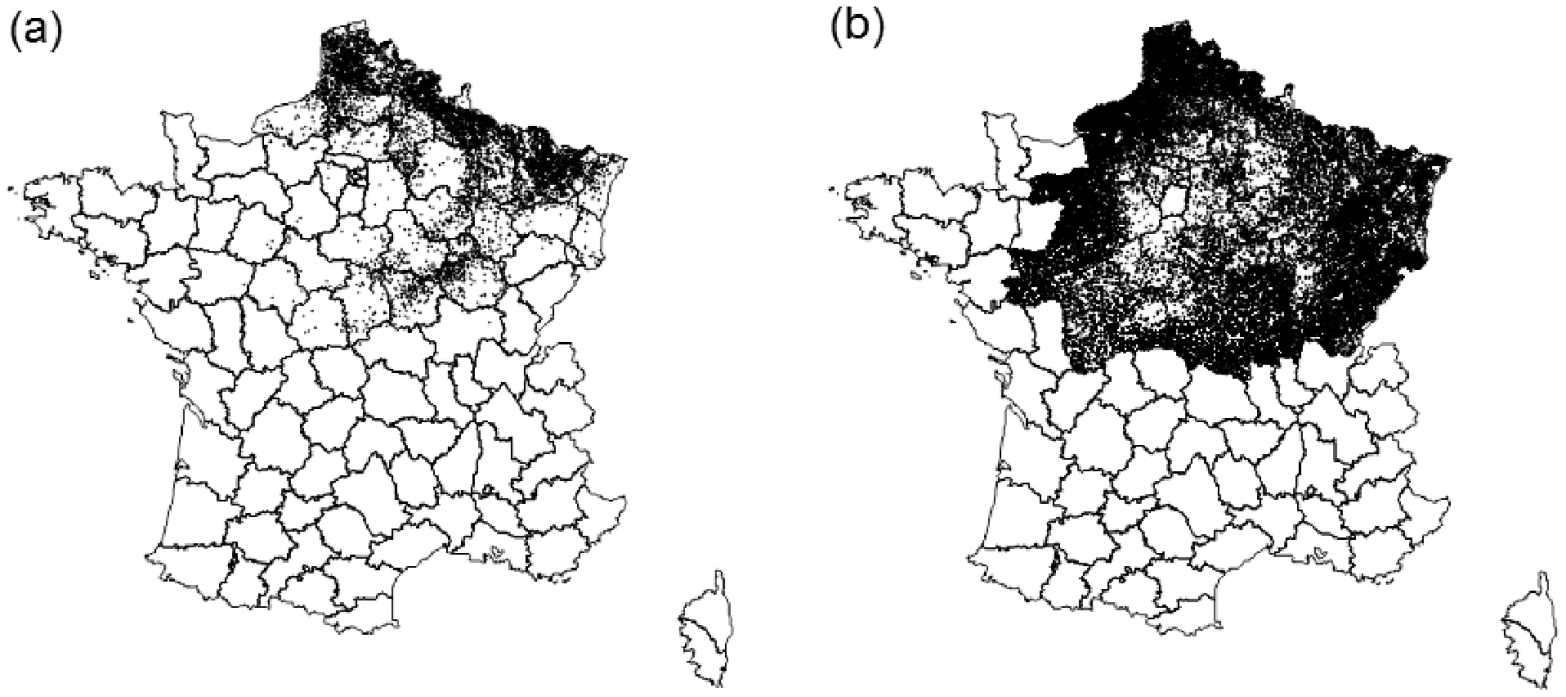
Geographical location of cattle herds in the outbreak area: (a) 8,313 case herds reported after clinical signs of Bluetongue virus serotype 8 (BTV-8) in 2007, (b) 74,169 non-reported herds (e.g., herds with an interpolated date of BTV-8 exposure); 2007; France.

Cattle case herds reported after clinical signs in 2007 were located in the north-eastern part of France (Figure 1(a)). The overall 90-day-return-to-service rates were 56.9% for cows in case herds and 56.0% for cows in herds with uncertain BTV-8 status. The overall 90-day-return-to-service rate was 54.2% in the reference population and 54.3% for cows inseminated in 2005 in herds that were located in the 2007 outbreak area (Table 1).

**Table 1:** Return-to-service rates and distribution of cantons, herds and cows according to herds Bluetongue virus serotype 8 (BTV-8) exposure statuses, 2007, France.

	Numberof cantons	Numberof herds	Numberof cows	90-day-return-to-servicerates (%)
Reported case herds with clinical signs	408	2,646	43,786*	56.9
Non-reported herds located in the 2007 outbreak area	648	5,237	78,293*	56.0
Unexposed herds in 2007	312	9,485	211,578**	54.2
Herds in 2005 that were located in the 2007 outbreak area	715	8,215	126,362*	54.3

*Cows with first artificial insemination (AI) between 10 weeks before and 4 weeks after the observed date of clinical detection (case herds), the interpolated date of clinical detection (non-reported herds) and the transposed date in a year free of BTV-8 (herds in 2005 located in the 2007 outbreak area).

**Cows with first AI performed during the same period in the year than herds located in the outbreak area.

### Interpolated Date of Clinical Signs Detection for Herds with Uncertain BTV-8 Status

**Figure 2:**
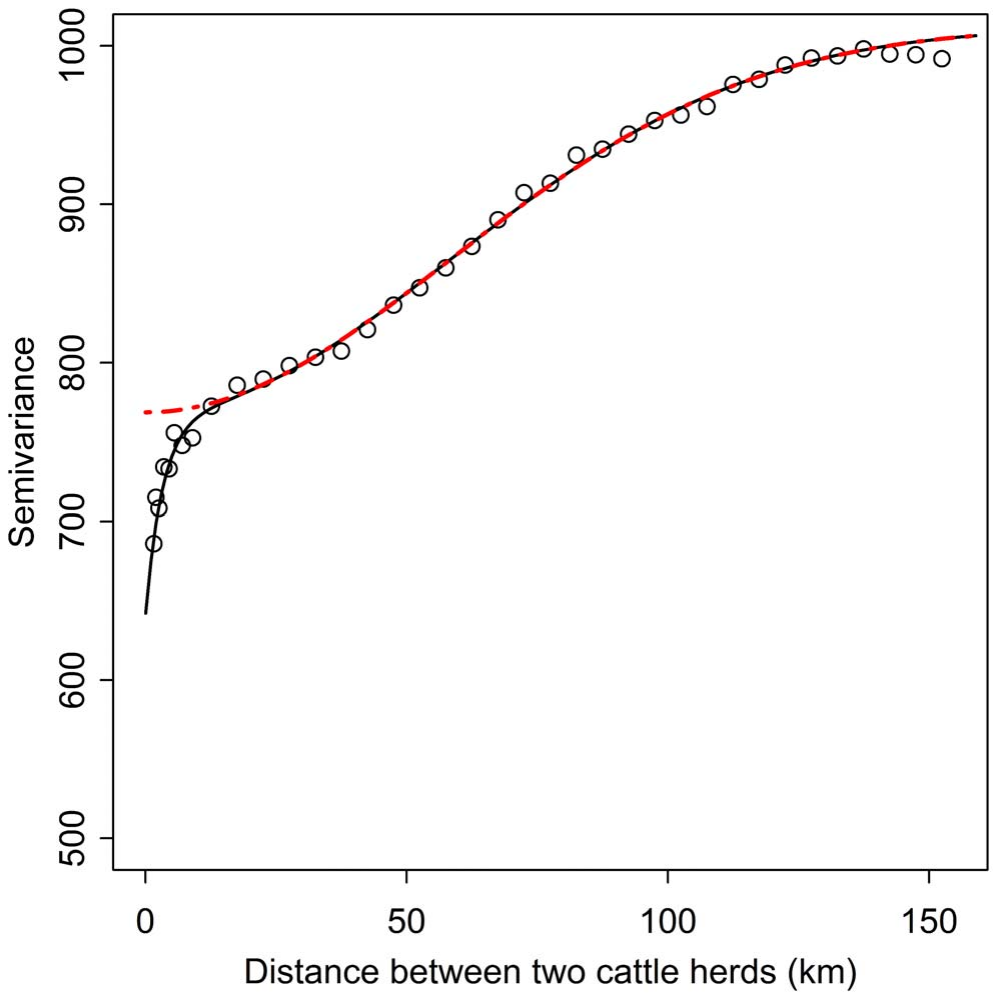
Experimental variogram of the observed dates of detection of Bluetongue virus serotype 8 clinical signs of reported case herds (dots) and the fitted nested model of semivariogram (solid black line) which is the sum of a nugget effect, an exponential and a Gaussian variogram model. The gaussian component which is kept for kriging is shown in red dashed line.

**Figure 3:**
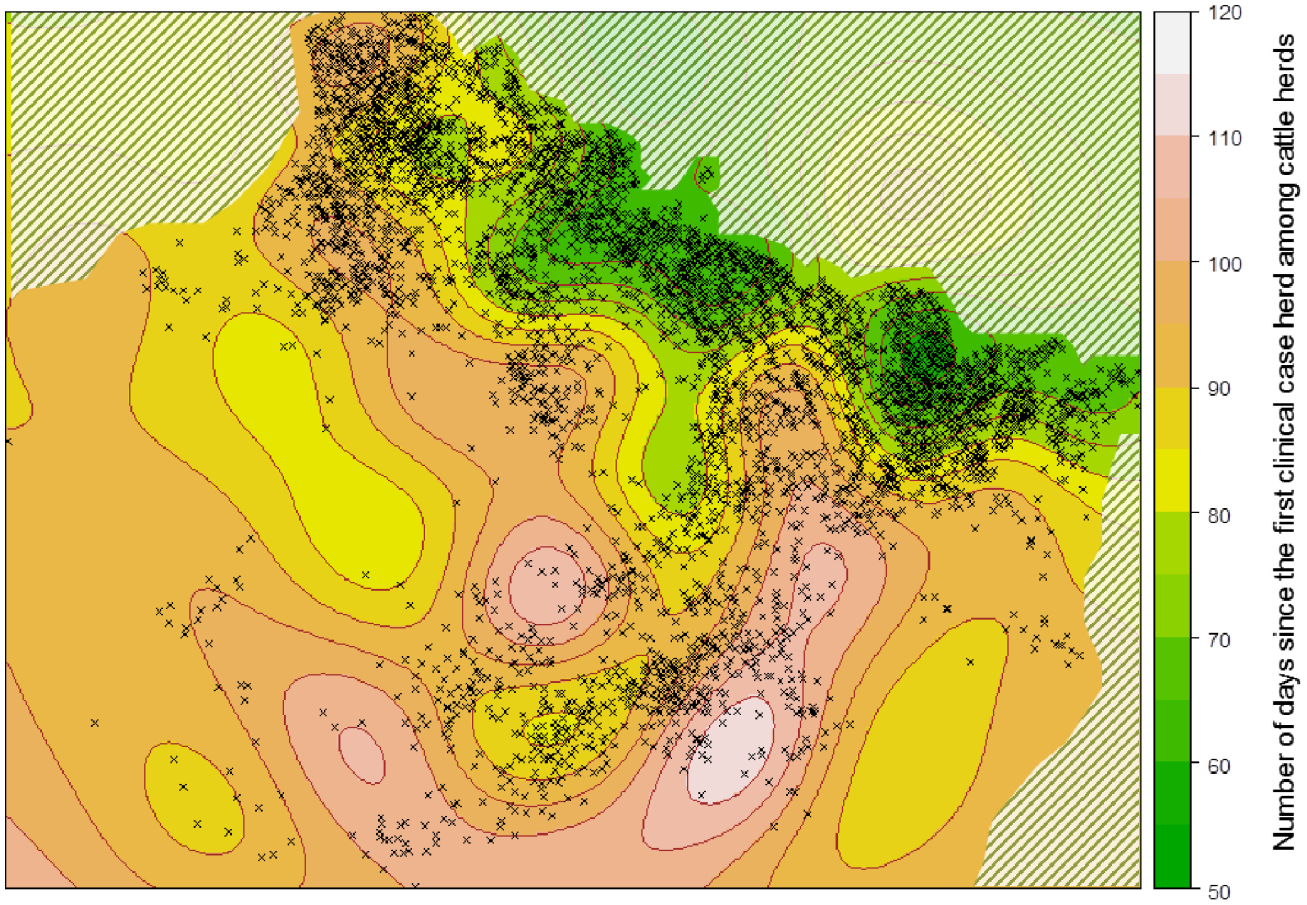
Kriging map of the dates of detection of Bluetongue virus serotype 8 clinical signs, expressed as a number of days since the first clinical case herd during the 2007 epizootic in France (31^st^ July 2007), and location of reported case herds (black crosses). The hatched areas correspond to regions with no data.

Figure 2 shows the experimental semivariogram of the observed dates of clinical signs detection in case herds. Some case herds located in the same municipality were detected at different periods of the epizootic. These point pairs had thus a large semivariance, giving a pure random term (nugget effect) of 640 day^2^ and a fitted exponential semivariogram model with a semi-variance of 127 day^2^ and a range of 9.9 km. The fitted gaussian semivariogram model had a semi-variance of 243 day^2^ for a scale parameter (sd) of 82 km that is equivalent to an effective range of about 160 km. The fitted nested semivariogram model, plotted in black in Figure 2, shows the suitability of the fitted model for all distances larger than 10 km. The Gaussian component of the variogram model, in red dashed line, was used to map mid-to-long-range variation by Universal Kriging, filtering short range variation (5 to 10 km) and semivariance due to location uncertainty inside the municipality level. Figure 3 shows the location of the 8,313 cattle herds used as the data sample and the predicted values of the kriging model for the dates of clinical detection of the disease in the outbreak area. Predicted dates of clinical suspicion were expressed as a number of days since the first clinical case detected the 31^st^ July 2007 among cattle herds.

### Under-reporting Evidenced Using Performance Data

**Figure 4:**
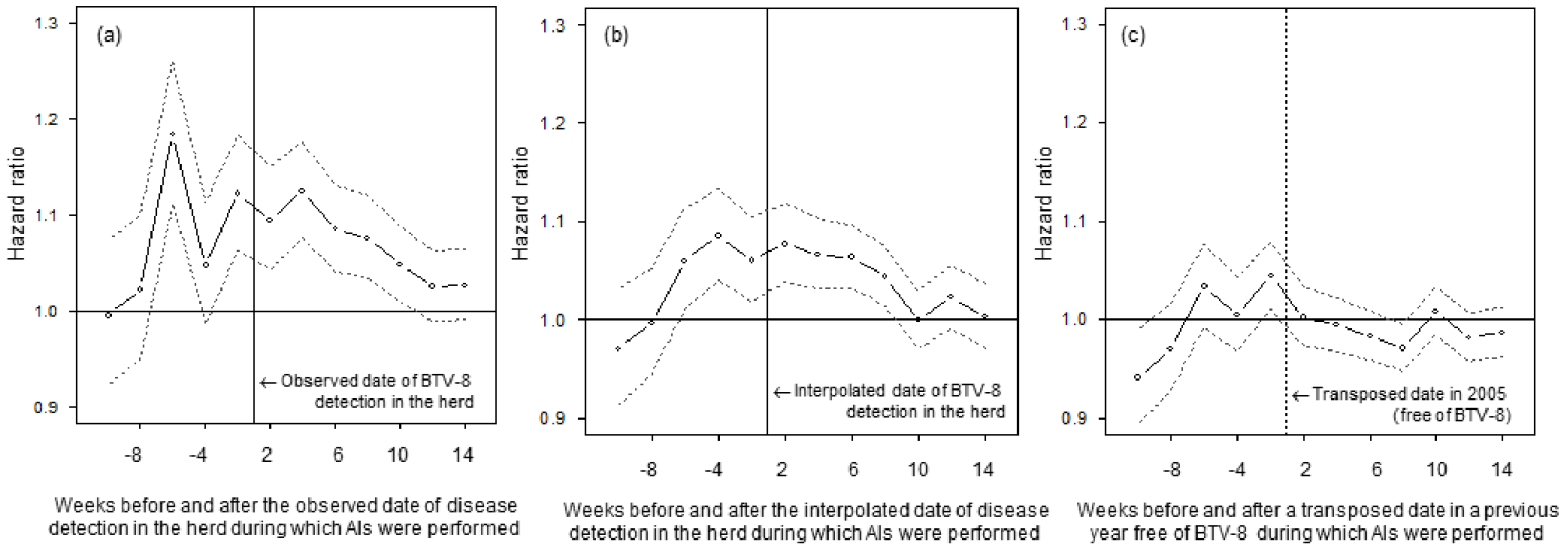
Hazard Ratio (HR) of 90-d-return-to-service before and after (a) the date of Bluetongue virus serotype 8 (BTV-8) clinical detection for case herds reported during the 2007 epizootic, (b) the interpolated date of BTV-8 clinical detection for non-reported herds located in the 2007 outbreak area, (c) the transposed date in a previous year free of BTV (2005) for herds in 2005 located in the 2007 outbreak area, France.

As expected, exposure to BTV-8 at the time were clinical signs were observed was associated with an increase of 90-d-return-to-service rate for cows in case herds (Figure 4-a). The period of fertility decrease corresponded to AIs performed between 6 weeks before to 10 weeks after the date of clinical detection (HR between 1.05 and 1.18). More interestingly, for cows in herds with uncertain BTV-8 status, an increase of the 90-day-return-to-service rate was also found (Figure 4-b). The period of decreased fertility corresponded to AIs performed between 6 weeks before and 8 weeks after the interpolated date (HR between 1.04 and 1.08). Finally, for cows inseminated in 2005 in herds that were located in the 2007 outbreak area, only a very slight increase of 90-day-return-to-service rate was observed for AIs performed 2 weeks before the date in a previous year free of BTV-8 (HR = 1.04) (Figure 4-c). These fertility decreases corresponded to an increase of 5.2 and 3.0 percentage points of 90-day-return-to-service for cows in case herds and cows in herds with uncertain BTV-8 status, respectively.

The following adjustment variables were significantly associated with a risk of 90-day-return-to-service: lactation number, milk yield, Protein:Fat ratio and calving-to-AI interval. The risk was higher for multiparous cows and for cows with a higher milk yield. In contrast, the risk was lower for cows with a higher Protein:Fat ratio and for cows with a higher calving-to-AI interval (Table 2).

**Table 2:** Effect of adjustment variables on risk of 90-day-return-to-service for cows which underwent artificial insemination (AI) between 10 weeks before and 14 weeks after the observed date of Bluetongue virus serotype 8 (BTV-8) detection (2,646 case herds), the interpolated date of BTV-8 clinical detection (5,237 non-reported herds) and the transposed date in a year free of BTV-8 (8,215 herds in 2005 located in the 2007 outbreak area).

Variable and classes	Number of AI	HR	95% CI*
Lactation number			
1	179,508	1	*Ref*
2	120,951	1.10	[1.09–1.12]
3	77,463	1.12	[1.11–1.13]
4 or more	82,137	1.21	[1.19–1.22]
Peak milk yield (category)#			
1	58,772	0.94	[0.93–0.95]
2	115,175	0.97	[0.96–0.98]
3	140,240	1	*Ref*
4	96,807	1.04	[1.03–1.05]
5	49,065	1.10	[1.08–1.11]
Protein:Fat ratio			
>0; ≤0.58	47,239	1.04	[1.02–1.05]
>0.58; ≤0.66	93,475	1.03	[1.02–1.04]
>0.66; ≤0.75	159,567	1	*Ref*
>0.75; ≤0.83	105,573	0.98	[0.97–0.99]
>0. 83; ≤1.5	54,205	0.97	[0.96–0.99]
Calving-to-AI interval (days)			
>35; ≤50	35,134	1.25	[1.23–1.27]
>50; ≤62	85,366	1.13	[1.12–1.15]
>62; ≤80	133,327	1.07	[1.05–1.08]
>80; ≤102	109,056	1	*Ref*
>102; ≤125	55,126	0.95	[0.94–0.97]
>125; ≤150	26,909	0.92	[0.90–0.94]
>150; ≤180	14,366	0.88	[0.86–0.91]
Month of service§			
May	16,198	0.92	[0.90–0.95]
June	16,167	0.89	[0.87–0.92]
July	21,238	0.95	[0.93–0.97]
August	34,268	0.96	[0.94–0.98]
September	46,444	1	*Ref*
October	71,011	1.01	[0.99–1.02]
November	99,904	0.97	[0.95–0.99]
December	86,194	0.98	[0.96–1.01]
January	45,041	0.98	[0.96–0.99]
February	23,594	1.01	[0.99–1.03]

*95% Confidence interval.

# Classes were constituted according to the distribution of the milk yield production and the lactation number.

§ Month of service in 2007 for cows in herds located in the 2007 outbreak area; month of service in 2005 for cows inseminated in 2005 in herds located in the 2007 outbreak area.

The unexposed population was composed of 211,578 cows in 9,485 herds, 2005–2007, France.

## Discussion

The results indicate that fertility data can be used to evidence the under-reporting of cases during an epizootic. Indeed, an decrease in fertility was found for cows in non-reported herds located in the 2007 outbreak area in France, corresponding to 60% of the effect for cows in case herds. Only a very slight decrease in fertility was found when comparing two populations of cows in unexposed herds, indicating that the episodes of fertility decreases quantified in this study were likely attributable to BTV-8 exposure. Moreover, the fertility decreases were precocious relative to the date of clinical suspicion.

The magnitude and duration of the episode of fertility decrease for cows in non-reported herds suggested that some of these herds have been infected by the virus during 2007. If assuming a similar average effect of infection between case herds and non-reported herds, around 60% of non-reported herds located in the 2007 outbreak area would have been infected (if the effect is lower in these herds, the percentage would be greater). This result indicates that production losses in non-reported infected herds could contribute to an important part of the total burden of the epizootic.

Results found in this study are consistent with other publications. A follow-up survey of 50 dairy herds in Belgium conducted between December 2007 and February 2008 indicated that only 20 herds were officially notified as BTV outbreaks whereas all had been infected with BTV-8. The authors indicated that similar discrepancies occurred in all provinces [2]. A study conducted in the Netherlands in 2007 quantified an increase in mortality of dairy cows in non-reported herds located in areas exposed to BTV-8 [12]. This effect corresponded to an increase of 1.11 (95% CI: 1.08–1.13) of the mortality rate ratio for cows >1 year while for cows in confirmed herds, the mortality rate ratio was equal to 1.41 (95% CI: 1.22–1.63). These results suggested that some of the herds that were not notified in exposed areas probably had suffered from BTV-8 infection. In our study, the fertility decrease in non-reported herds was relatively greater, in term of magnitude of effect, than the excess of mortality found in non-reported herds in the study of Santman-Berends [12]. An explanation could be that cow mortality lead more easily to the suspicion of BTV-8 exposure than a decrease of fertility. Moreover, the delay between virus exposure and the reproductive event observed made it difficult to associate them. There were probably less infected herds not reported that experienced an excess of mortality due to BTV-8 exposure than those which experienced a decrease of fertility for some cows.

Fertility could vary among regions due to differences in herd management and/or local weather conditions. The unexposed population considered in this study was composed of herds located in regions unexposed to the virus in 2007. A difference of fertility – independently of the exposure – between exposed and unexposed regions could bias the quantification of the effect of BTV-8 exposure. To check whether such a difference existed, the fertility before the beginning of BTV-8 epizootic, in 2005, was compared between the 2 regions exposed and unexposed. To do so, the same Cox model using the adjustment variables previously described was considered. No difference was shown, indicating that fertility was comparable before the exposure between the 2 regions. Therefore, the decreased fertility evidenced can be attributable to BTV-8 exposure and not to differences in herd management and/or local weather conditions. Finally, it was also check that there was no difference when comparing fertility between 2005 and 2007 in the exposed region.

The evidence of under-reporting of cases during an epizootic in the case of BTV-8 outbreak could be generalized to pathogens that affect cattle’s performance. In cattle, most pathogens, including zoonotic agents such as *Coxiella burnetii* [13], [14] or Rift Valley fever virus [15], have detrimental effects on cow’s performance. These effects can either be due to the tropism in genital tracts or indirectly due to the decreasing food intake associated with clinical signs.
